# Association Between Guideline-Discordant Prostate Cancer Imaging Rates and Health Care Service Among Veterans and Medicare Recipients

**DOI:** 10.1001/jamanetworkopen.2018.1172

**Published:** 2018-08-17

**Authors:** Danil V. Makarov, Shannon Ciprut, Dawn Walter, Matthew Kelly, Heather T. Gold, Xiao-Hua Zhou, Scott E. Sherman, Ronald Scott Braithwaite, Cary Gross, Steven Zeliadt

**Affiliations:** 1 Department of Urology, New York University School of Medicine, New York; 2 Department of Population Health, New York University School of Medicine, New York; 3 VA New York Harbor Healthcare System, New York University School of Medicine, New York; 4 Robert F. Wagner Graduate School of Public Service, New York University, New York; 5 Cancer Institute, New York University School of Medicine, New York; 6 Department of Biostatistics, University of Washington, Seattle; 7 Health Services Research and Development, Department of Veterans Affairs Medical Center, Seattle, Washington; 8 Cancer Outcomes Policy and Effectiveness Research Center, Yale University School of Medicine, New Haven, Connecticut; 9 Fred Hutchinson Cancer Research Center, Seattle, Washington

## Abstract

**Importance:**

Prostate cancer imaging rates appear to vary by health care setting. With the recent extension of the Veterans Access, Choice, and Accountability Act, the government has provided funds for veterans to seek care outside the Veterans Health Administration (VA). It is important to understand the difference in imaging rates and subsequent differences in patterns of care in the VA vs a traditional fee-for-service setting such as Medicare.

**Objective:**

To assess the association between prostate cancer imaging rates and a VA vs fee-for-service health care setting.

**Design, Setting, and Participants:**

This cohort study included data for men who received a diagnosis of prostate cancer from January 1, 2004, through March 31, 2008, that were collected from the VA Central Cancer Registry, linked to administrate claims and Medicare utilization records, and the Surveillance, Epidemiology, and End Results Program database. Three distinct nationally representative cohorts were constructed (use of VA only, use of Medicare only, and dual use of VA and Medicare). Men older than 85 years at diagnosis and men without high-risk features but missing any tumor risk characteristic (prostate-specific antigen, Gleason grade, or clinical stage) were excluded. Analysis of the data was completed from March 2016 to February 2018.

**Exposures:**

Patient utilization of different health care delivery systems.

**Main Outcomes and Measures:**

Rates of prostate cancer imaging were analyzed by health care setting (Medicare only, VA and Medicare, and VA only) among patients with low-risk prostate cancer and patients with high-risk prostate cancer.

**Results:**

Of 98 867 men with prostate cancer (77.4% white; mean [SD] age, 70.26 [7.48] years) in the study cohort, 57.3% were in the Medicare-only group, 14.5% in the VA and Medicare group, and 28.1% in the VA-only group. Among men with low-risk prostate cancer, the Medicare-only group had the highest rate of guideline-discordant imaging (52.5%), followed by the VA and Medicare group (50.9%) and the VA-only group (45.9%) (*P* < .001). Imaging rates for men with high-risk prostate cancer were not significantly different among the 3 groups. Multivariable analysis showed that individuals in the VA and Medicare group (risk ratio [RR], 0.87; 95% CI, 0.76-0.98) and VA-only group (RR, 0.79; 95% CI, 0.67-0.92) were less likely to receive guideline-discordant imaging than those in the Medicare-only group.

**Conclusions and Relevance:**

The results of this study suggest that patients with prostate cancer who use Medicare rather than the VA for health care could experience more utilization of health care services without an improvement in the quality of care.

## Key Points

**Question:**

What is the association between the type of health care delivery system and guideline-discordant prostate cancer imaging rates?

**Findings:**

In this cohort study that included data from 98 867 US men with a diagnosis of prostate cancer, Medicare beneficiaries were significantly more likely to receive guideline-discordant prostate cancer imaging than men treated only at the Veterans Health Administration.

**Meaning:**

Increased reliance on fee-for-service Medicare vs the Veterans Health Administration health system for prostate cancer treatment could be associated with the receipt of more unnecessary and potentially harmful tests.

## Teaser

This cohort study compares receipt of National Comprehensive Cancer Network (NCCN) guideline–discordant prostate cancer imaging (radionuclide, CT, or MRI) to stage incident prostate cancer among men being treated in the Veterans Health Administration system vs those receiving Medicare-covered services vs a combination of the two.

## Introduction

Reducing guideline-discordant prostate cancer staging imaging is an important national priority. Minimizing guideline-discordant imaging for men with low-risk prostate cancer is listed as a primary focus for reducing inefficient health care utilization in the Choosing Wisely campaign.^1,2,3^ Within the context of the Veterans Health Administration (VA) system, the Veterans Access, Choice, and Accountability Act, also known as the Choice Act, was passed in 2014 for the goal of reducing wait times for veterans seeking access to specialized health care services by providing funds for patients to seek care outside the VA.^4^ Changes in policy, such as the Choice Act, have unclear implications for the quality and cost-effectiveness of care that patients receive.

Prostate cancer imaging rates appear to vary among veterans depending on patients’ ability to seek care outside of the VA health system. Earlier research found higher rates of guideline-discordant prostate cancer imaging among VA patients with low-risk prostate cancer who used Medicare services than among those with no Medicare utilization.^5^ Within the VA, physicians typically receive a set salary that does not include financial incentives to provide more health care services.^6^ Outside the VA, the fee-for-service model used in Medicare and in most health care systems in the United States may encourage provision of more health care services^7^ because of direct physician reimbursement^8,9^ and patient self-referral. Earlier qualitative work found that physicians practicing at the VA are cognizant of this incentive difference between VA and non-VA practice and acknowledge that their own ordering behavior varies by setting.^10^ Many of these additional services that are associated with potential financial incentives may have limited efficacy or even be quantifiably unnecessary.^11^ Specifically, it is unclear whether a difference in care patterns among veterans seeking care only through the VA vs through the VA and Medicare would also apply to Medicare patients with no access to the VA.

The aim of this study was to directly assess the association between quality of health care within the VA health system vs a fee-for-service system (Medicare) by comparing rates of guideline-discordant and guideline-concordant imaging among patients with prostate cancer. To do this, we categorized patients into 1 of 3 groups: those who received health care through a fee-for-service system (the Medicare-only group), those who received health care through an integrated health system (the VA-only group), and those who received health care through a combination of the 2 systems (VA with some Medicare use; the VA and Medicare group).

Outside the VA health system, there may be higher rates of guideline-discordant prostate cancer imaging, suggesting a trade-off of resources for quality of care in a fee-for-service setting. If the rates of guideline-discordant prostate cancer imaging are actually lower outside the VA health system, it is possible that there is a problem in terms of quality of care in an integrated health system. We hypothesized that men with prostate cancer who used Medicare only would have the highest rate of guideline-discordant imaging, that those who used both health systems would have the next highest rate, and that those who used the VA only would have the lowest rate. The results of this study may help policy makers understand the implications of particular health care policies, such as the Choice Act, and highlight areas for improvement in the cost-effectiveness and quality of health care for veterans.

## Methods

We conducted a retrospective cohort study to compare rates of prostate cancer staging imaging among distinct health care settings. This study was approved by the institutional review boards of VA New York Harbor Healthcare and VA Puget Sound Healthcare Systems with a waiver of informed consent to include review of patient records. We used the VA Central Cancer Registry (VACCR), linked to administrate claims and Medicare utilization records, and the Surveillance, Epidemiology, and End Results Program (SEER) database to compose our cohorts according to the Strengthening the Reporting of Observational Studies in Epidemiology (STROBE) reporting guideline.^12,13^

We created 3 nationally representative cohorts of men who received a diagnosis of prostate cancer between 2004 and 2008: veterans who received a diagnosis at the VA, men whose diagnoses were found in SEER Medicare claims, and veterans who received a diagnosis at the VA and chose to receive subsequent care at private facilities using Medicare insurance.^5^ The VA patients were identified through the VA Information Resource Center Corporate Data Warehouse using inpatient, outpatient, surgery, and vital measures tables. Medicare patients were identified from the SEER Medicare database, which includes inpatient (MedPAR), outpatient, and physician claims. To identify dual users, we searched for any Medicare claims for veterans in our VA cohort and included these claims in our cohort data.^14,15^ The dual user group comprised VA patients who were eligible for Medicare benefits; there is no mechanism in place for Medicare patients to be referred to the VA for care. Of note, the VA sometimes outsources patient care to community health care professionals when the equipment or staff required to provide a specialized service is not available in a local VA medical center and the patient is unable to travel.^16,17^ Veterans who receive health care services at private facilities in this way, known as fee basis services in the VA, may have images obtained in the same facilities as Medicare users. Although we included these privately obtained services in our analysis, we classified these patients as part of the VA-only cohort because their imaging was directed solely by VA health care professionals. For all 3 groups, we identified prostate cancer diagnoses and associated care using *International Classification of Diseases, Ninth Revision* (*ICD-9*) diagnosis and procedure codes (92.14, 92.18, 88.01, and 88.95) and *Current Procedural Terminology/Healthcare Common Procedure Coding System* codes (78306, 78315, 78102, 78103, 78104, 72191, 72192, 72193, 72194, 74150, 74160, 74170, 74175, 72198, 74185, 72195, 72196, 72197, 74181, 74182, and 74183).

The study population consisted of men who received a diagnosis of prostate cancer between January 1, 2004, and March 31, 2008. We included patients younger than 85 years at diagnosis with a pathologically confirmed, registry-documented diagnosis, thus eliminating men with a diagnosis at autopsy or on the death certificate. We excluded men who died within 3 months after diagnosis and men without utilization of any health benefits in months 2 to 6 after diagnosis, a situation suggesting complete departure from the health care system.^5^ In addition, men who received a diagnosis at facilities with a prostate cancer diagnosis volume less than 25 cases per year were eliminated because of the unreliability of clinical data from such institutions.^18^ We also excluded men without high-risk features but missing any tumor risk characteristic (prostate-specific antigen [PSA], Gleason grade, or clinical stage), because we could not confirm the risk classification of these men.

Our primary dependent variable of interest was receipt of any of the following imaging studies: radionuclide bone scan, computed tomography (CT), or magnetic resonance imaging (MRI). Guideline concordance was determined on the basis of National Comprehensive Cancer Network (NCCN) recommendations for imaging to stage incident prostate cancer.^19^ Receipt of bone scan was considered to be guideline discordant unless a patient had any of the following high-risk characteristics: clinical stage T3 or higher, Gleason score 8 or higher, and PSA level of 20 ng/mL or higher. Receipt of CT or MRI was considered to be guideline discordant unless the patient had a 20% or higher risk of positive lymph nodes, estimated from the Partin tables.^20^ Men who met the requirements for bone scan, CT, or MRI were classified as having high-risk prostate cancer, whereas all other men were classified as having low-risk prostate cancer. Two exceptions were made to these rules regarding guideline concordance of imaging. Receipt of CT was considered to be guideline concordant for patients with low-risk prostate cancer if they were undergoing radiation therapy, because CT may have been used for treatment planning rather than for disease staging.^21^ In addition, receipt of bone scan by a patient with low-risk prostate cancer was considered to be guideline concordant if the patient had a diagnosis of spinal or pathologic fractures in the 3-month period before prostate cancer diagnosis or until 6 months after prostate cancer diagnosis.^22^ Patients with low-risk prostate cancer were classified as guideline discordant if they received an inappropriate bone scan, CT, or MRI. Patients with high-risk prostate cancer were classified as guideline concordant if they received a bone scan, CT, or MRI. It is possible that patients received multiple scans; we did not measure surplus guideline-discordant imaging among patients with low-risk prostate cancer or surplus guideline-concordant imaging among patients with high-risk prostate cancer for this study.

The VA and Medicare claims were reviewed for the period beginning 3 months before diagnosis until cancer treatment initiation, death, or 6 months after diagnosis, whichever was earliest. Patient treatment for prostate cancer was identified using *ICD-9* diagnosis codes and categorized on the basis of the earliest treatment date as radical prostatectomy, radiation, or other treatment. Other treatment included androgen deprivation, cryosurgery, and watchful waiting (ie, no treatment found).

Data also included age at diagnosis, race, marital status, geographical region of the institution where the diagnosis was given, clinical stage, PSA level at diagnosis, Gleason score at diagnosis, and diagnosis year. In addition, we analyzed claims for the year before prostate cancer diagnosis to calculate an unweighted Elixhauser comorbidity score.^23^ Median household income and proportion of the population with a college degree were identified for each patient’s county of residence on the basis of 2010 US Census data.^24^ The institution where a diagnosis was given was categorized on the basis of the average number of prostate cancer cases diagnosed per year during the study period as high volume (>99 cases), medium volume (60-99 cases), or low volume (<60 cases).^5^

### Statistical Analysis

We created a multivariable logistic regression model to determine the association between all described covariates and the receipt of imaging, stratified by high-risk and low-risk cancer and clustered by region. All covariates were considered to be theoretically important and thus remained in the model regardless of their level of statistical significance. Because the VA cohort included men younger than 65 years and the Medicare cohort did not, we performed a sensitivity analysis that included only men 65 years and older. We report adjusted risk ratios (RRs) computed from the fitted logistic regression model. Statistical analyses were performed using Stata software (version 12.0; StataCorp). All *P* values were 2-sided with statistical significance at α = .05. Analysis of the data was completed between March 2016 and February 2018.

## Results

The cohort included 98 867 men with incident prostate cancer (mean [SD] age, 70.26 [7.48] years). Table 1 shows that the majority of the sample (57.3%) were in the Medicare-only group, followed by 28.1% in the VA-only group and 14.5% in the VA and Medicare group. Reflecting current nationwide patterns,^5,25^ the majority of the cohort (69.8%) were categorized as having low-risk prostate cancer. The overall study sample was predominantly white (77.4%), followed by black (17.4%), other races (2.8%), and missing or unknown race (2.3%). The largest age group in the cohort was 75 to 85 years (28.9%), followed by 70 to 74 years (25.9%), 65 to 69 years (25.1%), and 64 years and younger (20.0%). Because we limited the Medicare group to men who were age qualified, this group had no members younger than 65 years. Most of the men in the cohort (62.9%) were married. For all variables across the 3 groups, χ^2^ tests revealed statistically significant differences. Results of the sensitivity analysis limited to men 65 years and older revealed no significant differences in the magnitude or direction of the odds ratios from the primary analysis.

**Table 1.** Demographic and Clinical Characteristics of Men With Incident Prostate Cancer

Characteristic	No. (%) of Men			
	VA only (n = 27 811)	VA and Medicare (n = 14 385)	Medicare Only (n = 56 671)	Total (N = 98 867)
Prostate cancer imaging group				
Low-risk prostate cancer	20 066 (72.2)	9963 (69.3)	38 973 (68.8)	69 002 (69.8)
High-risk prostate cancer	7745 (27.8)	4422 (30.7)	17 698 (31.2)	29 865 (30.2)
Clinical stage				
T1	17 626 (63.4)	8817 (61.3)	31 203 (55.1)	57 646 (58.3)
T2NOS	1237 (4.5)	747 (5.2)	13 806 (24.4)	15 790 (16.0)
T2A	3046 (11.0)	1592 (11.1)	2722 (4.8)	7360 (7.4)
T2B	1280 (4.6)	719 (5.0)	1159 (2.1)	3158 (3.2)
T2C	3489 (12.6)	2012 (14.0)	4562 (8.1)	10 063 (10.2)
T3	626 (2.3)	280 (2.0)	1658 (2.9)	2564 (2.6)
T4	209 (0.8)	100 (0.7)	605 (1.1)	914 (0.9)
Missing	298 (1.1)	118 (0.8)	956 (1.7)	1372 (1.4)
Gleason grade				
<7	12 845 (46.2)	6163 (42.8)	23 698 (41.8)	42 706 (43.2)
3 + 4	7408 (26.6)	3684 (25.6)	14 229 (25.1)	25 321 (25.6)
4 + 3	2794 (10.1)	1613 (11.2)	6390 (11.3)	10 797 (10.9)
≥8	4566 (16.4)	2802 (19.5)	11 179 (19.7)	18 547 (18.8)
Missing	198 (0.7)	123 (0.9)	1175 (2.1)	1496 (1.5)
PSA level, ng/mL				
0-4	3172 (11.4)	1543 (10.7)	6763 (11.9)	11 478 (11.6)
>4 to 10	16 103 (57.9)	8077 (56.2)	30 894 (54.5)	55 074 (55.7)
>10 to 20	4070 (14.6)	2387 (16.6)	9375 (16.5)	15 832 (16.0)
>20	4315 (15.5)	2285 (15.9)	7702 (13.6)	14 302 (14.5)
Missing	151 (0.5)	93 (0.7)	1937 (3.4)	2181 (2.2)
Race				
Black	8014 (28.8)	2752 (19.1)	6429 (11.3)	17 195 (17.4)
White	18 834 (67.7)	11 041 (76.8)	46 694 (82.4)	76 569 (77.4)
Other	283 (1.0)	142 (1.0)	2372 (4.2)	2797 (2.8)
Missing	680 (2.5)	450 (3.1)	1176 (2.1)	2306 (2.3)
Age, y				
<65	17 792 (64.0)	1999 (13.9)	0	19 791 (20.0)
65-69	3817 (13.7)	3750 (26.1)	17 291 (30.5)	24 858 (25.1)
70-74	3198 (11.5)	4356 (30.3)	18 094 (31.9)	25 648 (25.9)
≥75	3004 (10.8)	4280 (29.8)	21 286 (37.6)	28 570 (28.9)
Marital status				
Married	13 784 (49.6)	9025 (62.7)	39 402 (69.5)	62 211 (62.9)
Single, divorced, or widowed	13 872 (49.9)	5311 (36.9)	11 780 (20.8)	30 963 (31.3)
Missing	155 (0.6)	49 (0.3)	5489 (9.7)	5693 (5.8)
Medical comorbidities				
0	7344 (26.4)	3381 (23.5)	42 436 (74.9)	53 161 (53.8)
1-2	9217 (33.1)	4585 (31.9)	13 209 (23.3)	27 011 (27.3)
≥3	11 250 (40.5)	6419 (44.6)	1026 (1.8)	18 695 (18.9)
Treatment				
Watchful waiting and/or hormone therapy	11 327 (40.7)	5928 (41.2)	22 934 (40.5)	40 189 (40.6)
Prostatectomy	7913 (28.5)	2646 (18.4)	13 784 (24.3)	24 343 (24.6)
Radiation therapy	8571 (30.8)	5811 (40.4)	19 953 (35.2)	34 335 (34.7)
Hospital volume category, cases per y				
<60	2739 (9.9)	2019 (14.0)	24 922 (44.0)	29 680 (30.0)
60-99	6656 (23.9)	3506 (24.4)	9830 (17.4)	19 992 (20.2)
>99	18 416 (66.2)	8860 (61.6)	10 826 (19.1)	38 102 (38.5)
Missing	0	0	11 093 (19.6)	11 093 (88.8)
Census tract per capita income, $				
<25 000	3261 (11.7)	1386 (9.6)	4250 (7.5)	8897 (9.0)
25 000-34 999	8716 (31.3)	4929 (34.3)	10 219 (18.0)	23 864 (24.1)
35 000-44 999	7953 (28.6)	4243 (29.5)	11 875 (21.0)	24 071 (24.3)
45 000-54 999	3692 (13.3)	1799 (12.5)	9776 (17.3)	15 267 (15.4)
>55 000	3098 (11.1)	1505 (10.5)	20 210 (35.7)	24 813 (25.1)
Missing	1091 (3.9)	523 (3.6)	341 (0.6)	1955 (2.0)
Census tract population with ≥4 y of college, %				
<10	4792 (17.2)	2438(17.0)	9206 (16.2)	16 436 (16.6)
10 to <20	12 069 (43.4)	6612 (46.0)	16 278 (28.7)	34 959 (35.4)
20 to <30	5064 (18.2)	2395 (16.7)	9863 (17.4)	17 322 (17.5)
≥30	4468 (16.1)	2212 (15.4)	20 409 (36.0)	27 089 (27.4)
Missing	1418 (5.1)	728 (5.1)	915 (1.6)	3061 (3.1)
Year of diagnosis				
2004	6575 (23.6)	2723 (18.9)	14 253 (25.2)	23 551 (23.8)
2005	6036 (21.7)	3171 (22.0)	13 657 (24.1)	22 864 (23.1)
2006	6365 (22.9)	3592 (25.0)	14 451 (25.5)	24 408 (24.7)
2007	7262 (26.1)	4030 (28.0)	14 310 (25.3)	25 602 (25.9)
2008	1573 (5.7)	869 (6.0)	0	2442 (2.5)

Abbreviations: PSA, prostate-specific antigen; VA, Veterans Health Administration.

### Men With Low-Risk Prostate Cancer

Among men with low-risk prostate cancer, more men in the Medicare-only group received at least 1 imaging test for staging (52.5%) compared with the VA and Medicare group (50.9%) and VA-only group (45.9%) (*P* < .001) (Table 2). The Medicare-only group was also least likely to receive guideline-concordant care (53.1%) vs the VA and Medicare group (56.4%) and VA-only group (60.6%) (*P* < .001) (Table 2). Results of the multivariable model showed that, compared with the Medicare-only reference group, being a VA and Medicare group patient (RR, 0.87; 95% CI, 0.76-0.98) or a VA-only group patient (RR, 0.79; 95% CI, 0.67-0.92) was associated with reduced guideline-discordant imaging (Table 3).^26^ For men with low-risk prostate cancer, all clinical markers were significantly associated with receipt of imaging. Clinical stage of T2B (RR, 1.25; 95% CI, 1.17-1.33) or T2C (RR, 1.21; 95% CI, 1.15-1.27), Gleason score of 7 or higher (Gleason score 3 + 4: RR, 1.23; 95% CI, 1.17-1.28; Gleason score 4 + 3: RR, 1.38; 95% CI, 1.30-1.46), and PSA level greater than 10 ng/mL (RR, 1.52; 95% CI, 1.39-1.64) were associated with increased receipt of guideline-discordant imaging. In addition, an increased level of comorbidity was associated with greater risk of guideline-discordant imaging, whereas living in a county with higher educational levels was associated with lower rates of imaging (Table 3).

**Table 2.** Imaging Use Among Men Who Received a Diagnosis of Incident Prostate Cancer^a^

Imaging Modality	No. (%) of Men		
	VA Only (n = 27 811)	VA and Medicare (n = 14 385)	Medicare Only (n = 56 671)
Men with low-risk disease characteristics			
Guideline-concordant care	12 161 (60.6)	5620 (56.4)	20 686 (53.1)
Any imaging	9214 (45.9)	5068 (50.9)	20 455 (52.5)
Bone scan	6474 (32.3)	3552 (35.7)	16 014 (41.1)
CT	6769 (33.7)	3694 (37.1)	13 516 (34.7)
MRI	363 (1.8)	369 (3.7)	2287 (5.9)
Men with high-risk disease characteristics			
Guideline-concordant care	5322 (68.7)	3181 (71.2)	11 814 (66.8)
Any imaging	5833 (75.3)	3494 (79.0)	13 583 (76.8)
Bone scan	5166 (66.7)	3111 (70.4)	12 454 (70.4)
CT	4488 (58.0)	2580 (58.3)	9645 (54.5)
MRI	270 (3.5)	219 (5.0)	1363 (7.7)

Abbreviations: CT, computed tomography; MRI, magnetic resonance imaging; VA, Veterans Health Administration.

^a^ Within 3 months before prostate cancer diagnosis and up to 6 months after diagnosis.

*P* < .001 for all associations by χ^2^ test.

**Table 3.** Adjusted Risk Ratios of Receipt of Imaging Staging Test Associated With Clinical and Demographic Factors Among Men With Incident Prostate Cancer, Stratified by Imaging Indication and Clustered by VA Region

Variable	Risk Ratio (95% CI)	
	Low-Risk Prostate Cancer	High-Risk Prostate Cancer
Insurance group		
Medicare only	1 [Reference]	1 [Reference]
VA only	0.79 (0.67-0.92)^a^	1.00 (0.95-1.06)
VA and Medicare	0.87 (0.76-0.98)^a^	1.04 (0.98-1.09)
Clinical stage		
T1	1 [Reference]	1 [Reference]
T2NOS	1.03 (0.96-1.09)	1.08 (1.05-1.11)^a^
T2A	1.07 (1.00-1.15)	0.99 (0.92-1.05)
T2B	1.25 (1.17-1.33)^a^	1.07 (1.02-1.13)^a^
T2C	1.21 (1.15-1.27)^a^	1.08 (1.04-1.12)^a^
T3	NA	1.06
T4	NA	0.98
Missing	NA	1.03
Gleason grade		
<7	1 [Reference]	1 [Reference]
3 + 4	1.23 (1.17-1.28)^a^	1.24 (1.15-1.35)^a^
4 + 3	1.38 (1.30-1.46)^a^	1.32 (1.20-1.43)^a^
≥8	NA	1.45 (1.31-1.58)
Missing	NA	1.14
PSA level, ng/mL		
0-4	1 [Reference]	1 [Reference]
>4 to 10	0.95 (0.92-0.99)^a^	0.97 (0.93-1.01)
>10 to 20	1.52 (1.39-1.64)^a^	1.06 (1.02-1.11)^a^
>20	NA	1.1
Missing	NA	0.91
Race		
Black	1 [Reference]	1 [Reference]
White	0.94 (0.86-1.01)	0.98 (0.95-1.02)
Other	0.97 (0.81-1.12)	1.02 (0.96-1.08)
Missing	0.85 (0.73-096)	0.89 (0.82-0.97)^a^
Age, y		
<65	1 [Reference]	1 [Reference]
65-69	0.98 (0.92-1.03)	1.01 (0.98-1.04)
70-74	1.00 (0.94-1.07)	1.04 (1.00-1.07)^a^
≥75	1.02 (0.95-1.09)	0.99 (0.95-1.02)
Marital status		
Married	0.98 (0.95-1.01)	1.00 (0.98-1.03)
Single, divorced, or widowed	1 [Reference]	1 [Reference]
Missing	0.97 (0.92-1.03)	1.07 (1.02-1.11)
Medical comorbidities		
0	1 [Reference]	1 [Reference]
1-2	1.08 (1.05-1.11)^a^	1.03 (1.01-1.06)^a^
≥3	1.12 (1.07-1.16)^a^	1.01 (0.99-1.03)
Hospital volume category, cases per y		
<60	1 [Reference]	1 [Reference]
60-99	0.90 (0.81-0.98)^a^	1.00 (0.95-1.05)
>99	0.93 (0.84-1.01)	1.03 (0.98-1.08)
Missing	0.78 (0.72-0.83)	0.86 (0.82-0.90)
Census tract per capita income, $		
<25 000	1 [Reference]	1 [Reference]
25 000-34 999	0.90 (0.79-1.02)	0.96 (0.91-1.02)
35 000-44 999	0.95 (0.95-0.96)^a^	1.01 (1.01-1.01)^a^
45 000-54 999	0.97 (0.96-0.97)^a^	1.02 (1.01-1.02)^a^
>55 000	1.05 (1.04-1.05)^a^	1.03 (1.03-1.04)^a^
Missing or unknown	1.11 (1.09-1.12**)**	1.02 (1.02-1.02)
Census tract population with ≥4 y of college, %		
<10	1 [Reference]	1 [Reference]
10 to <20	0.99 (0.94-1.05)	1.00 (0.97-1.03)
20 to <30	0.94 (0.86-1.01)	1.00 (0.96-1.04)
≥30	0.91 (0.83-1.00)^a^	1.01 (0.97-1.05)
Missing	0.89 (0.79-0.99)	0.97 (0.90-1.05)
Year of diagnosis		
2004	1 [Reference]	1 [Reference]
2005	1.01 (0.98-1.04)	1.05 (1.02-1.09)^a^
2006	1.03 (0.99-1.07)	1.09 (1.05-1.13)^a^
2007	1.03 (0.98-1.08)	1.13 (1.09-1.18)^a^
2008	0.96 (0.88-1.04)	1.18 (1.10-1.26)^a^

Abbreviations: NA, not available; PSA, prostate-specific antigen; VA, Veterans Health Administration.

^a^ *P* < .05, determined using adjusted risk ratios from multivariate logistic regression models with delta method standard errors.^26^

### Men With High-Risk Prostate Cancer

The proportion of men with high-risk prostate cancer who received at least 1 imaging test was similar among the 3 health care delivery systems, ranging from 75.3% to 79.0% (*P* < .001) (Table 2). The rate of guideline-concordant imaging among patients with high-risk prostate cancer was also similar among the health care delivery systems, with the VA and Medicare group having a slightly higher percentage of guideline-concordant patients with high-risk prostate cancer (71.2%), followed by the VA-only group (68.7%) and the Medicare-only group (66.8%) (Table 2). The multivariate model confirmed that, for men who received a diagnosis of high-risk prostate cancer, where they sought care was not significantly associated with receipt of guideline-concordant imaging. Gleason score of 7 or higher (Gleason score 3 + 4: RR, 1.24 95% CI, 1.15-1.35; Gleason score 4 + 3: RR, 1.32; 95% CI, 1.20-1.43; and Gleason score ≥8: RR, 1.45; 95% CI, 1.31-1.58), greater median county income (>$55 000: RR, 1.03; 95% CI, 1.03-1.04), and later year of diagnosis (2005: RR, 1.05; 95% CI, 1.02-1.09; 2006: RR, 1.09; 95% CI, 1.05-1.13; 2007: RR, 1.13; 95% CI, 1.09-1.18; and 2008: RR, 1.18; 95% CI, 1.10-1.26) had a statistically significant association with receipt of guideline-concordant imaging among men with high-risk prostate cancer (Table 3).

## Discussion

In this study, we analyzed rates of prostate cancer staging imaging among men with a diagnosis of prostate cancer in 3 types of health care delivery settings. We found that men with low-risk disease were less likely to receive guideline-discordant staging imaging if they received a diagnosis and treatment in the VA exclusively compared with those who received care using Medicare or a combination of VA and Medicare services. Furthermore, dividing our study sample into 3 cohorts based on delivery system revealed a dose-response relationship, with men with low-risk prostate cancer in the VA-only group having the lowest likelihood of guideline-discordant imaging, those in the VA and Medicare group having the next highest likelihood of guideline-discordant imaging, and those in the Medicare-only group having the highest likelihood of guideline-discordant imaging. The VA and Medicare health systems differ in notable ways, including their patient demographics and institutional characteristics,^27,28,29^ but it is possible that the differing financial incentives for physicians between these 2 health care delivery systems contributed to significantly different risks of guideline-concordant imaging among men with low-risk cancer. Although differing financial incentives were associated with differences in the rates of guideline-discordant imaging between the 2 groups, the rate of guideline-discordant imaging among patients with low-risk prostate cancer in the VA was high (45.9%) compared with that in other health care delivery systems without financial incentives for overuse, such as in Sweden, where recent guideline-discordant imaging rates were as low as 3% after an intervention to bring imaging practices in line with guidelines.^30^ This finding suggests that factors in addition to financial incentives, such as physician education, culture, and habituation, could also contribute to guideline-discordant prostate cancer imaging rates in the United States.

Disease characteristics were also associated with the receipt of imaging, whether guideline discordant for men with low-risk disease or guideline concordant for men with high-risk disease. Presentation with higher clinical stage, Gleason grade, or PSA level was associated with men with low-risk prostate cancer receiving presumably unwarranted imaging tests. For men with high-risk disease, only increased Gleason grade was associated with guideline-concordant imaging. These findings suggest that physicians find it difficult to adhere to evidence-based heuristics when faced with test results that approach high-risk levels, even when these test results are not sufficient to categorize the patient as having high-risk disease.^19^ There is thus an opportunity to reinforce physician knowledge and confidence in nomograms, such as the updated Partin tables,^31^ when making decisions regarding imaging of patients with prostate cancer.

Among men with high-risk disease, we found that the likelihood of receiving guideline-concordant imaging increased with each calendar year. This finding is encouraging and suggests that health care professionals became more aware across the study period of the importance of imaging in accordance with guidelines to stage more advanced cases of prostate cancer. Because this pattern did not occur among men with low-risk prostate cancer, findings suggest that physician education efforts may need to be targeted specifically with regard to the patients with low-risk prostate cancer.

There are numerous indications that guideline-discordant imaging among men with low-risk prostate cancer is still a salient problem. A 2017 systematic review identified 14 articles on overuse of imaging among patients with low-risk prostate cancer, representing data from 2004 to 2012.^32^ In the previous 10 years, there were several published efforts to curb guideline-discordant imaging, some of which have been successful, like the Michigan Urological Surgery Improvement Collaborative,^33^ and others which have been less so.^34^ Guideline-discordant staging imaging for low-risk prostate cancer continues to be regarded as a Choosing Wisely priority. This study provides important baseline data, highlights a prevalent practice pattern targeted for deimplementation, and may serve as a benchmark for more updated analyses to understand where quality improvement efforts may be successful and where they might be failing.

### Strengths and Limitations

Our study is strengthened by the large size of our study sample and the meticulous formation of each of the 3 delivery system cohorts. Because our study included men who received diagnosis and treatment using the VA and Medicare, which are 2 of the largest health care systems in the United States,^35,36^ our results are both reliable and generalizable. By creating a distinct cohort of men who used both the VA and Medicare, we were able to uniquely investigate the relative effectiveness of this commonly used approach to health care utilization.

We conducted our assessment of imaging utilization solely through analyses of claims data, and thus there was an inherent possibility of misclassification bias. Situations in which imaging was warranted for a patient with low-risk prostate cancer because of clinical information that was not noted specifically in claims may have been misclassified as guideline discordant. In addition, both Medicare and the VA have reported possible problems with data reliability regarding clinical stage and PSA level.^37^ Incorrect clinical stage or PSA level could have caused patients to be classified in the incorrect risk group, although this type of broad misclassification is rare. It is also possible that health care professionals have dual appointments and treat patients with prostate cancer both at the VA and at an affiliated medical center. Based on an earlier qualitative study, individual health care professionals have expressed that they alter their imaging practices on the basis of health care setting.^10^ Because we did not study the breakdown of health care professionals with dual appointments among the cohorts, the effect of this practice would be unclear in our results and should be further studied.

Our analysis data ended in 2008 and would benefit from an update to include more recent years. However, we carefully constructed these cohorts from multiple data sources and data sets, which is a time-consuming process with lags in availability, to uncover these important results. Veterans Health Administration regulatory requirements and new data server requirements to obtain updated data would require more than $50 000 in additional funding support just to obtain the data, before consideration of any analyst time, which is why we have not pursued establishing this data set as a repository. In addition, the approval timeline would be approximately 6 to 9 months, further impeding feasibility. The assembly of multiple data sources, even within the VA, requires complex data use agreements, which is one of the reasons that the analytic data set that we have assembled, although somewhat historical, is uniquely valuable.

## Conclusions

In this analysis, which merged 2 large prostate cancer cohorts (VACCR and SEER-Medicare), we found that veterans treated in a VA-only setting received the least amount of guideline-discordant imaging among patients with low-risk prostate cancer, without any significant difference in imaging utilization for patients with high-risk prostate cancer, for whom the imaging was necessary. In addition, although veterans who sought care through both the VA and Medicare would seem to be the most likely to experience the most imaging overuse because of the potential for fragmented care in 2 systems, patients seen in the Medicare-only setting had the most guideline-discordant imaging among patients with low-risk prostate cancer. These results reveal important differences between integrated and fee-for-service health systems regarding guideline concordance and quality of care. The results also suggest that patients using the Choice Act are likely to experience more utilization of care without a guarantee of improved quality of care. Future research to improve guideline-concordant care for prostate cancer imaging should consider and explore varying contexts and the role of unique settings of different health care systems. In addition, future studies should consider the cost implications of guideline-discordant imaging and the potential savings from an effort to align practice with evidence.
